# Aorto-venous fistula between an abdominal aortic aneurysm and an aberrant renal vein: a case report

**DOI:** 10.1186/1752-1947-4-255

**Published:** 2010-08-08

**Authors:** Mélanie Faucherre, Nader Haftgoli-Bakhtiari, Markus Menth, Julien Gaude, Beat Lehmann

**Affiliations:** 1 Department of Internal Medicine, Cantonal Hospital, Fribourg, 1700, Switzerland; 2 Emergency Department, Cantonal Hospital, Fribourg, 1700, Switzerland; 3 Department of Surgery, Cantonal Hospital, Fribourg, 1700, Switzerland; 4 Department of Radiology, Cantonal Hospital, Fribourg, 1700, Switzerland

## Abstract

**Introduction:**

The potential complications of an abdominal aortic aneurysm include rupture, compression of surrounding structures, thrombo-embolic events and fistula. The most common site of arterio-venous fistula is the inferior vena cava. Fistula involving a renal vein is particularly uncommon.

**Case presentation:**

This report describes a 54-year-old Caucasian woman who was admitted to the emergency department with fatigue, severe dyspnea and bilateral lower limb edema. In the first instance this anamnesis suggested possible heart failure. In fact, our patient presented with multi-organ system failure due to a fistula between an infra-renal aortic aneurysm and an aberrant retro-aortic renal vein.

**Conclusions:**

To our knowledge, this is the first report of a woman with a fistula between an infra-renal aortic aneurysm and an aberrant retro-aortic left renal vein. Aorto-venous fistulas may be asymptomatic or may present with symptoms characteristic of arterio-venous shunting and/or aneurysm rupture. This type of fistula is a rare cause of heart failure. Clinical examination and imaging are essential for detection.

## Introduction

The most common complication of abdominal aortic aneurysm (AAA) is rupture. Direct ruptures into a nearby organ, such as the duodenum and the venous system remain very rare [1]. Fistula involving a renal vein is particularly uncommon [2].

Aorto-venous fistulas may be asymptomatic or may present with symptoms characteristic of arterio-venous shunting and/or aneurysm rupture [3]. Symptoms such as chest pain, signs of acute heart failure with or without electrocardiographic signs of acute coronary ischemia or a long history of chronic heart failure resistant to therapy are often present [1]. The classic triad of clinical symptoms and signs in the AAA patients with aorto-caval fistula are abdominal or back pain (or both), a pulsatile mass, and an abdominal bruit. In a review of 148 reported cases, Gilling-Smith *et al*. reported that this classic triad is present in only 63% [4]. The extent of the clinical manifestations of a fistula between an AAA and the venous system depends on the size, duration and location of the fistula [5].

This report describes a 54-year-old Caucasian woman who was admitted to the emergency department with fatigue, severe dyspnea and bilateral lower limb edema. In the first instance this anamnesis suggested possible heart failure. In fact, our patient presented with multi-organ system failure due to a fistula between an infra-renal aortic aneurysm and an aberrant retro-aortic renal vein.

## Case presentation

A 54-year-old Caucasian woman was referred to our emergency department for heart failure associated with dyspnea and bilateral lower limb edema, which had persisted for two months. Her past medical history is significant for hypertension and obesity (body mass index: 34 kg/m^2^).

**Figure 1:**
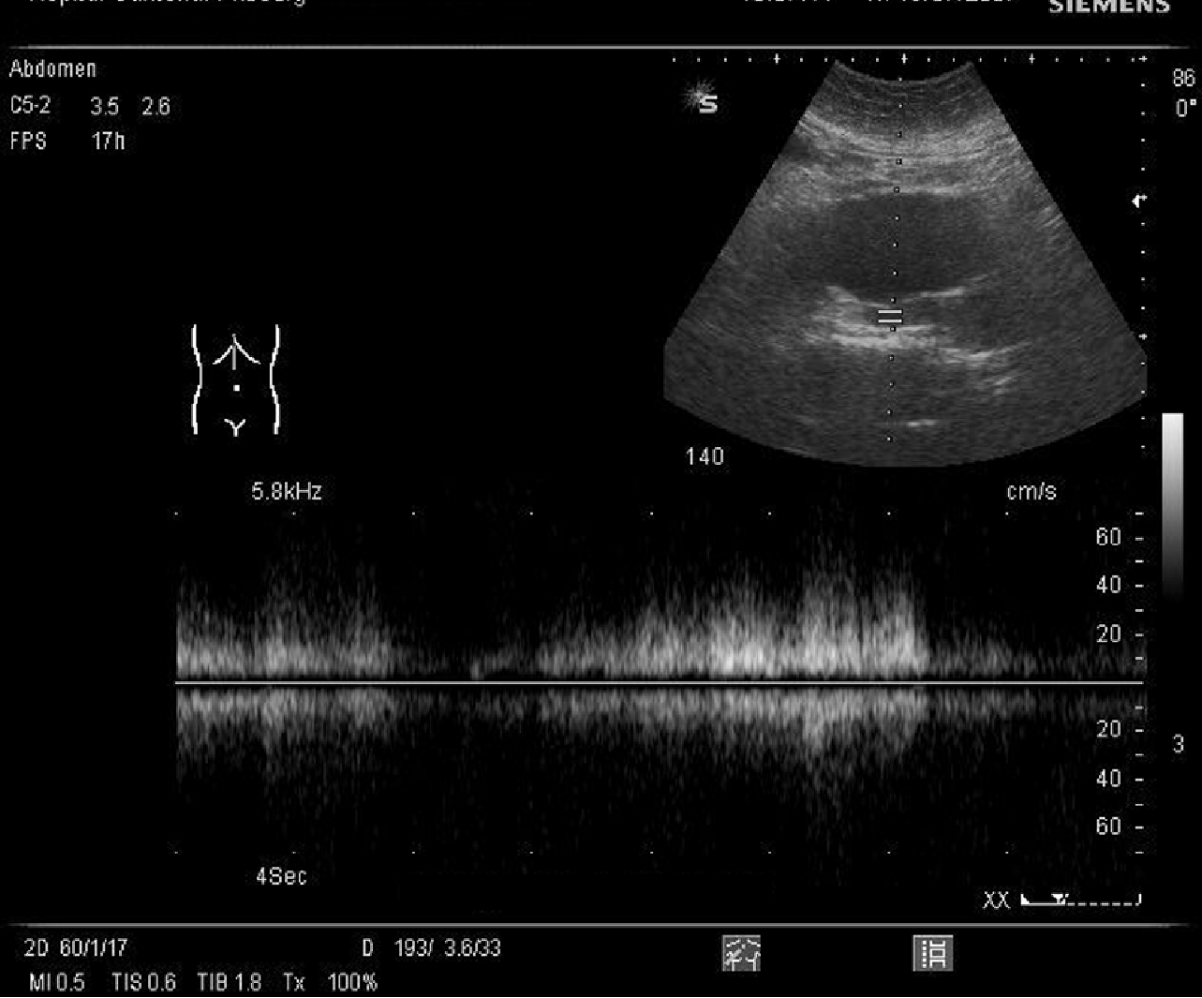
**Abdominal ultrasound at the emergency department demonstrating the presence of a vascular structure behind the abdominal aortic aneurysm with a mixed arterio-venous flow due to the arterio-venous fistula**.

**Figure 2:**
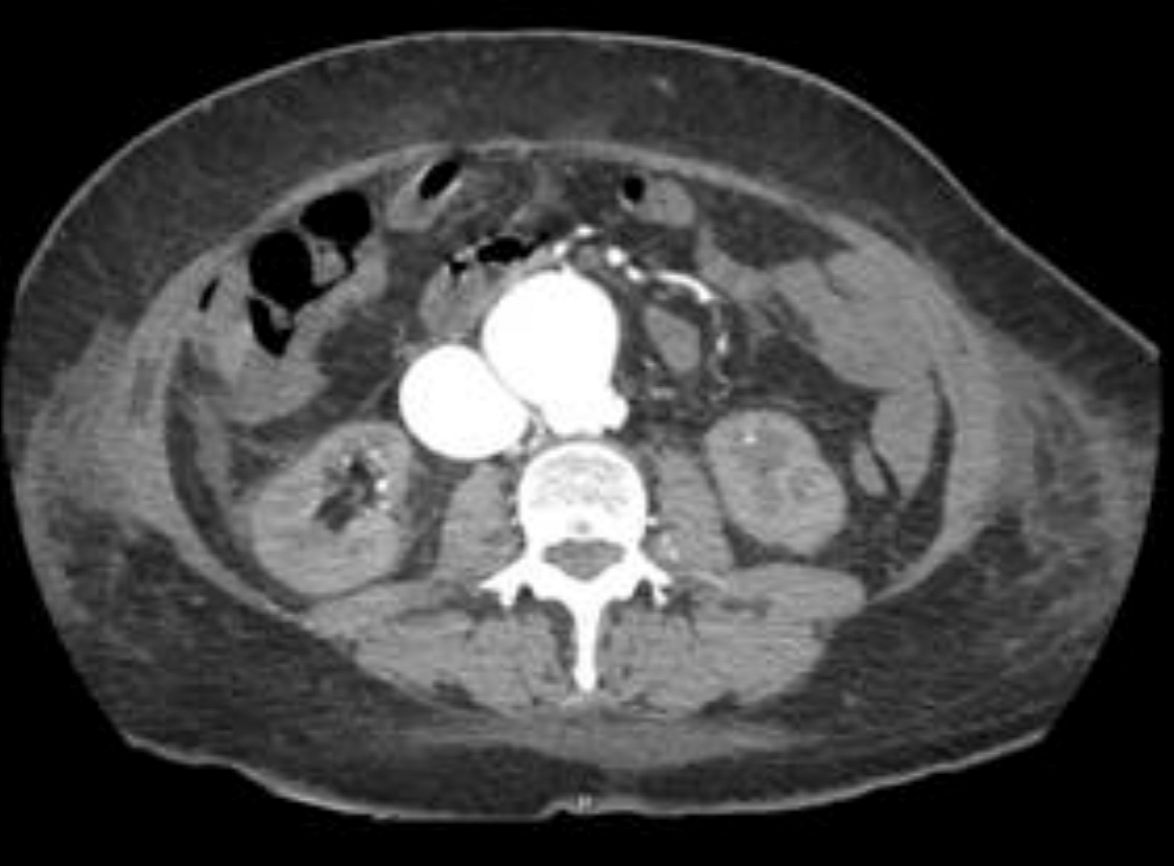
**Contrast CT scan at the emergency department confirming the fistula between AAA and the aberrant left renal vein**.

**Figure 3:**
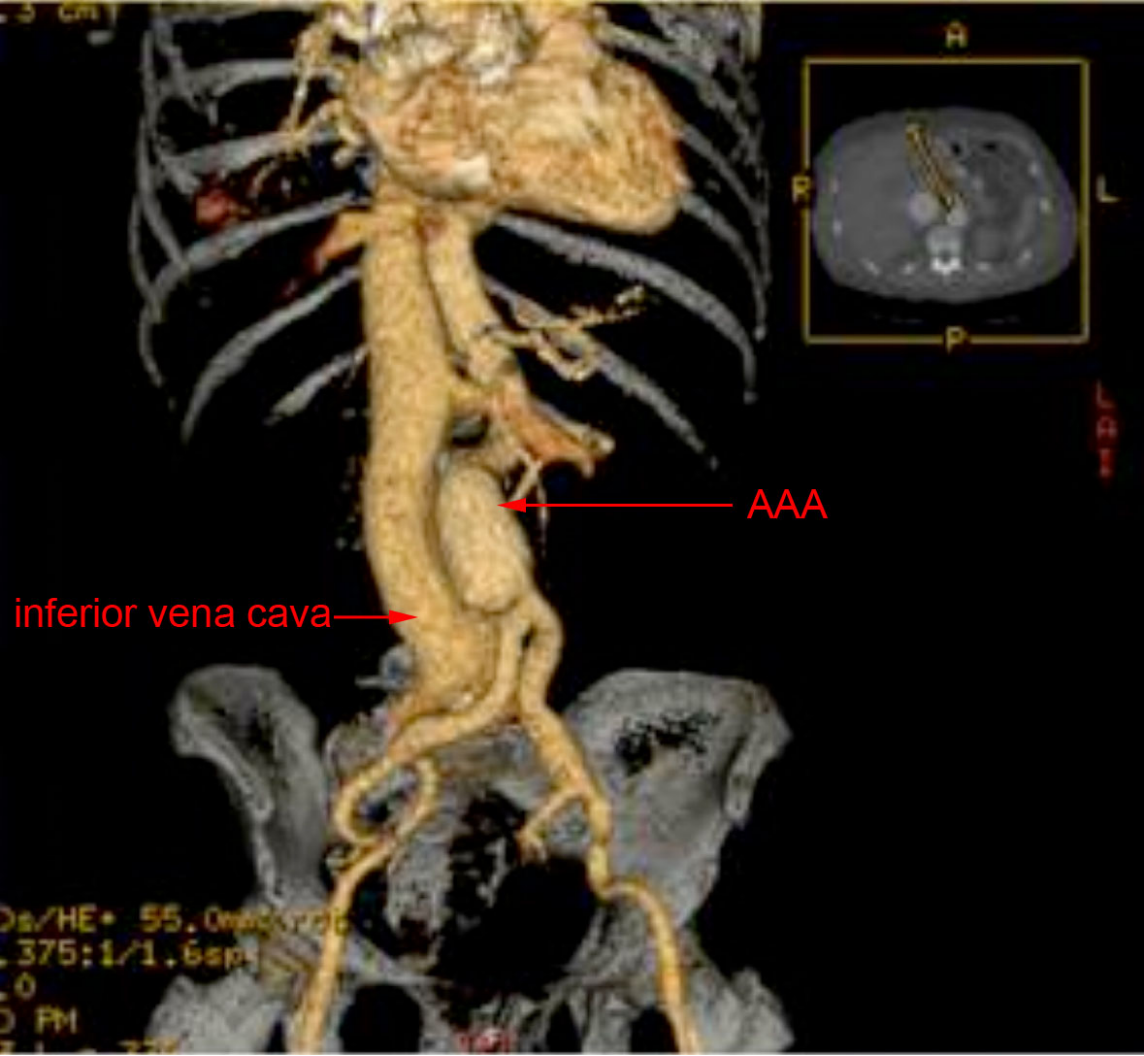
**3D contrast CT showing abdominal aortic aneurysm and the dilated inferior cava vein**.

On admission to hospital, her blood pressure was 120/70 mmHg and heart rate 90/min; there was a systolic murmur (3/6) which was predominant at the apex; distension of the jugular vein indicating elevation of central venous pressure and there was pitting edema of both legs. Thoracic percussion revealed a right basal dullness that was compatible with pleural effusion. These signs were suggestive of heart failure. On abdominal auscultation, a systolo-diastolic murmur was audible. Furthermore, we observed hematomas on both arms and legs. Ultrasound showed an AAA, a vascular structure behind the AAA, as well as a massively dilated inferior vena cava with arterial flow velocity features (Figure 1). The results of laboratory tests revealed liver dysfunction (aspartate aminotransferase (ASAT) 120 IU/L, reference value (rv): <40 U/L; bilirubin 51.1 μmol/L, rv: 3.1-18.8 μmol/L and lactate dehydrogenase (LDH) 979 IU/L, rv: <450 IU/L), renal failure (serum creatinin 241 μmol/L, rv: 50-95 μmol/L), thrombocytopenia (80 G/L, rv: 150-300 G/L) as well as coagulation disturbances (PT: 33%, rv: 70-100%; PTT: 42 s, rv: 26-35 s; fibrinogen: 0.7 g/L, rv: 2-4.5 g/L). A computed tomography (CT) scan confirmed a partially thrombosed AAA with a maximal antero-posterior diameter of 4.2 cm. A flush of intravenous contrast product was detected in the left (aberrant) renal vein immediately after injection due to a fistula between the AAA and the aberrant left renal vein (Figures 2 and 3).

Arteriovenous shunt resulted in an increase of venous return, pressure and volume with simultaneous fall in the peripheral resistances: heart rate, stroke volume, cardiac output and cardiac work increase as a physiological response to the overload. It induced hyperdynamic cardiac failure; this explains the perturbation of the liver and renal function [1]. Moreover, the increase of the renal venous pressure aggravated this dysfunction.

A xyphopubic laparotomy was performed on the same day. The surgeon clamped the aorta, both iliac arteries and the inferior vena cava upstream and downstream the retroaortic renal vein. The hematoma inside the aneurysm was removed. The retro-aortic left renal vein was ligated. The fistula was plugged with parietal tissue and a ligature. For the aneurysm, a straight silver graft (with a diameter of 16 mm) was interposed; the aorto-prosthetic and termino-terminal anastomoses were completed without complication. During the operation, the cell saver collected 6200 mL. A biopsy of the aneurysm wall was sent to a pathology institute; analysis revealed rare elastic fibers, a fibro-muscular hyperplasia of the tunica intima and atheroma. Microbiological analyses were negative. Her post-operative course was favorable with both liver and renal function tests returning to normal.

## Discussion

By definition, an AAA is present if there is a dilation of the abdominal aorta to a size 50% greater than the proximal normal segment or to a maximum aortic diameter greater than 3 cm. The overall prevalence of AAA ranges between 4 and 8% in men and is about 1% in women [6]. Risks factors for AAA are male sex [7], smoking, age greater than 65 years and a positive family history. Less important risk factors include established vascular disease, hypercholesterolemia, low HDL-cholesterol, hypertension and increased height [8]. Patients with connective tissue disorders (e.g. Marfan's syndrome) or vasculitis (e.g. Takayasu arteritis or giant-cell arteritis) are particularly at risk of developing an AAA. People with diabetes and women are at lower risk of developing AAA [8]. AAAs are often asymptomatic until rupture. The risk of rupture increases with the increasing diameter of the aneurysm.

Clinical examination and imaging are essential to detect AAA. The sensitivity of abdominal palpation [9] increases significantly with the diameter of the AAA. In a pooled analysis of 15 studies of abdominal palpation for AAA detection, the sensitivity ranged from 29% to 76% and the positive predictive value was about 43% [6]. Palpation of AAA appears to be safe and has not been reported to precipitate rupture. Screening abdominal ultrasound in asymptomatic individuals is an accurate test, with 95% sensitivity and near 100% specificity to detect aneurysms greater than 3.0 cm [8]. CT and magnetic resonance imaging provide high-resolution imaging of the aorta and determine proximal and distal boundaries of the aneurysm [6]. A fistula should be suspected if there is a flush of contrast product in a dilated venous system immediately after the injection.

The potential complications of AAA include rupture, fistulas, compression of surrounding structures, infections and thrombo-embolic events. The most common complication of AAA is rupture, either into the retroperitoneum or into the abdominal cavity. Direct rupture into a nearby organ, such as the duodenum or the venous system, or the infra-renal vena cava, renal vein or the primary iliac vein, remain very rare and is often discovered peri-operatively [1]. The most common site of arterio-venous fistula is the inferior vena cava; iliac and renal veins are only rarely affected. According to the literature, the incidence of aorto-caval fistulas is low, ranging from 0.22 to 6.04% of all AAA [10]. Fistulas involving a renal vein are particularly uncommon [2].

Aorto-venous fistulas may be asymptomatic or may present with symptoms characteristic of arterio-venous shunting and/or aneurysm rupture [3]. The typical clinical findings are abdominal, lumbar or flank pain, pulsatile abdominal mass with continuous abdominal bruit or thrill, signs of congestive heart failure and hematuria. Symptoms such as chest pain, signs of acute heart failure with or without electrocardiographic signs of acute coronary ischemia or a long history of chronic heart failure resistant to therapy are often present [1]. The classic triad of clinical symptoms and signs in AAA patients with aorto-caval fistula are abdominal or back pain (or both), a pulsatile mass, and an abdominal bruit. In a review of 148 reported cases, Gilling-Smith *et al*. reported that this classic triad is present in only 63% [4]. The extent of the clinical manifestations of a fistula between an AAA and the venous system depends on the size, duration and location of the fistula [5].

Retroaortic renal veins are found in 1.8% of autopsies. Signs and symptoms of aorto-renal vein fistulas are similar to those of ureteral colic, and form a unique group of patients with aorto-venous fistula. Left flank pain and hematuria are present in almost all reported cases. Heart failure is rare in this situation, which is presumably explained by the relatively small volume of fistula flow usually present [11].

## Conclusions

Early diagnosis is crucial in the management of aorto-renal vein fistulas. Acting on a high level of suspicion, a careful clinical examination, followed by imaging studies (ultrasound) can provide further information. Pre-operative diagnosis can be accomplished with the careful interpretation of CT scans that gives rapidly precise information. The results of surgical treatment for this condition have been favorable when pre-operative localization has been precise and the operative technique cautious [4]. Problems in the treatment of aorto-caval fistula include poor patient condition due to hemorrhagic shock, high-output heart failure, renal failure and intra-operative bleeding. Usually, cardiac and renal abnormalities are rapidly reversed after surgical closure of the fistula.

Arterio-venous fistula is a rare but well known cause of heart failure. A pulsatile abdominal mass and an abdominal murmur should be followed by imaging studies (ultrasound, CT scan), and a definitive diagnosis is usually made by CT scanning. Treatment is by surgical repair with a bifurcated graft, a straight tube graft, and endovascular aneurysm repair (EVAR). Usually, cardiac and renal abnormalities are rapidly reversed after surgical closure of the fistula.

## List of abbreviations

AAA: abdominal aortic aneurysm; ASAT: aspartate aminotransferase; CT: computed tomography; HDL: high-density lipoprotein; IU/L: international units per liter; LDH: lactate dehydrogenase; PT: prothrombin time; PTT: activated partial thromboplastin time; RV: reference value.

## Competing interests

The authors declare that they have no competing interests.

## Authors' contributions

MF and BL supervised the case at the emergency department, contributed to the literature research. MF wrote this case report with BL as a contributor. NH and MM contributed to analysis and interpretation of the clinical and radiological findings of the patient. JG interpreted the CT scan. All authors read critically and approved the manuscript.

## Consent

Written informed consent was obtained from the patient for the publication of this case report and any accompanying images. A copy of the written consent is available for review by the Editor-in-Chief of this journal.
